# Subclinical pulmonary embolism in first-line/induction therapy of lung cancer - a remaining clinically relevant adverse event

**DOI:** 10.3389/fonc.2026.1886848

**Published:** 2026-09-21

**Authors:** Kristin Mersmann, Martin Metzenmacher, Gregor Zaun, Marcel Wiesweg, Filiz Oezkan, Jane Winantea, Christian Taube, Dirk Theegarten, Martin Schuler, Lale Umutlu, Servet Bölükbas, Fabian Doerr, Daniel Messiha, Marcel Opitz, Wilfried EE Eberhardt, Johannes Wienker

**Affiliations:** 1 Department of Medical Oncology, West German Cancer Center, University Hospital Essen, Essen, Germany; 2 National Center for Tumor Diseases (NCT), NCT West, Essen, Germany; 3 Division of Thoracic Oncology, West German Cancer Center, University Medicine Essen - Ruhrlandklinik, Essen, Germany; 4 Department of Pulmonology, West German Cancer Center, University Medicine Essen - Ruhrlandklinik, Essen, Germany; 5 Department of Pathology, West German Cancer Center, University Hospital Essen, Essen, Germany; 6 Institute of Diagnostic and Interventional Radiology and Neuroradiology, West German Cancer Center, University Hospital Essen, University of Duisburg-Essen, Essen, Germany; 7 Department of Thoracic Surgery, University Medicine Essen – Ruhrlandklinik, Essen, Germany; 8 Department of Cardiology and Vascular Medicine, West German Heart and Vascular Centre, University of Duisburg-Essen, Essen, Germany

**Keywords:** chemotherapy, induction therapy, lung cancer, pulmonary embolism, thromboembolic complications

## Abstract

**Introduction:**

Pulmonary embolism (PE) at initial diagnosis and during first-line systemic treatment is clinically relevant in patients with lung cancer. However, real-world incidence of subclinical PE is not well established.

**Methods:**

Retrospective single-center analysis of patients at least 18 years old with non-small-cell lung cancer (NSCLC) or small-cell lung cancer (SCLC) who received first-line/induction systemic therapy. All patients underwent contrast-enhanced computed tomography (CT) of the thorax with bolus tracking for PE detection. Treatment response was assessed by restaging at weeks 6 and 12 after treatment initiation.

**Results:**

A total of 373 patients treated between 2019 and 2022 were included (261 NSCLC, 112 SCLC; 179 women and 194 men; median age 66 years [range, 22 to 86]; median smoking history 40 Pack Years [range, 0-150]). There was a predominance of locally advanced and metastatic disease (IB-IIB, n = 22; IIIA-C, n = 123; IVA-B, n = 228). Adenocarcinoma was the predominant histology among NSCLC cases (LUAD, n = 146), followed by squamous cell carcinoma and other NSCLC subtypes combined (n = 115); 112 patients had SCLC.

PE was detected in 61/373 patients, of whom 58 (95%) had subclinical PE. In NSCLC, body-mass-index (BMI) was independently associated with occurrence of PE (OR = 1.111, p = 0.005), while central tumor localization (OR 5.637, p = 0.028) and vascular infiltration (OR 7.683, p = 0.010) were significantly associated with PE in SCLC. All patients with PE received anticoagulants according to national and international guidelines and no PE-related deaths occurred.

**Conclusion:**

This single-center analysis demonstrates a high incidence of subclinical PE in newly diagnosed lung cancer patients and identifies clinical risk factors associated with PE development.

## Introduction

Pulmonary embolism (PE) is a relatively common event that strongly contributes to morbidity and mortality through mechanisms such as recurrent embolism and right heart failure (1, 2). Prognosis worsens for lung cancer patients who concurrently suffer from PE (3). Among all cancer types, lung cancer, particularly adenocarcinoma (LUAD), is associated with an increased risk of PE (3–5). To the best of our knowledge, subclinical PE is not uniformly defined in current guidelines. It generally refers to clinically asymptomatic PEs detected incidentally during imaging studies. In lung cancer patients, such findings frequently occur during staging examinations, likely due to the nonspecific clinical presentation of PE, which frequently overlaps with cancer-related symptoms such as pleuritic chest pain, dyspnea and hemoptysis (1, 6, 7). Hence, many PE cases in lung cancer patients remain asymptomatic and therefore underrecognized in this population (8).

Imaging studies for staging and response assessment in patients with lung cancer are standardized. Guidelines recommend FDG-PET/CT for staging and CT for follow-up. Most patients receive 4 to 5 courses of chemotherapy-based systemic treatment resulting in the following imaging time points (1): initial staging prior to treatment (2), interim response assessment after two to three cycles, and (3) post-therapeutic evaluation following the completion of systemic (induction) therapy (9, 10). Additional imaging studies are conducted to monitor maintenance therapy or at follow-up.

Although prophylactic anticoagulation is recommended for cancer patients particularly with immobility or recent surgery, its application remains individualized, balancing the elevated risk of venous thromboembolism against the bleeding risk (11–13). This individualized approach is reflected in current international guidelines (ASCO, ITAC and ESC Cardio-Oncology) (14–16). Specifically, the ESC guidelines recommend prophylactic anticoagulation with low-molecular-weight heparin (LMWH) for hospitalized patients with cancer and those requiring prolonged bed rest, whereas the ASCO guidelines emphasize that incidental PE should generally be treated in the same manner as symptomatic venous thromboembolism (VTE) (14, 16).

In lung cancer patients PE may arise from a complex interplay of patient characteristics, tumor biology, laboratory markers and treatment associated determinants. Various factors contribute to the thrombotic risk in lung cancer patients. Immobility due to cancer-related symptoms or surgery, along with patient-related factors such as obesity, play a critical role (6, 17). Beyond laboratory findings, tumor characteristics such as central localization and metastatic spread significantly influence PE risk (18). Centrally located tumors are more likely to obstruct pulmonary vessels, while regional and distant metastases, as well as higher number of metastatic sites, have all been associated with elevated PE incidence (19).

Treatment-related factors further contribute to thrombogenesis. Systemic platinum-based chemotherapies such as cisplatin have a particularly strong association with venous thromboembolism (3, 19–21). In recent years, targeted therapies and immune checkpoint inhibitors (ICIs) have become integral to lung cancer treatment (22, 23). Emerging evidence links ICIs to an increased PE risk, although this risk appears lower compared to cytotoxic chemotherapy (20, 24, 25).

This study aims to retrospectively evaluate clinical, laboratory and tumor-related risk factors for clinical and especially subclinical PE in lung cancer patients with platinum-based induction chemo/(immuno) therapy undergoing standard imaging for therapy monitoring. These findings may support improved risk stratification and targeted thromboprophylaxis in lung cancer patients.

## Methods

### Study design and population

We conducted a retrospective, single-center cohort study including consecutive patients with histologically confirmed lung cancer treated at the Division of Thoracic Oncology, University Medicine Essen - Ruhrlandklinik, Germany, between January 2019 and December 2022. Patients ≥18 years of age with available contrast-enhanced chest CT scans performed for routine staging and response assessment were eligible. Both non-small-cell lung cancer (NSCLC) and small-cell lung cancer (SCLC) patients across all disease stages were included. Patients with insufficient imaging quality or incomplete clinical data, as well as patients with previous anticoagulation treatment, were excluded.

Subclinical PE was defined as an incidental filling defect in the pulmonary arterial tree detected on contrast-enhanced CT performed for indications other than suspected PE, in the absence of documented PE-related symptoms. Incidental PE was identified on routine contrast-enhanced thoracic CT scans obtained for staging or scheduled treatment response assessment. Dedicated CT pulmonary angiography (CTPA) was reserved for patients with clinical suspicion of pulmonary embolism. All CT examinations were reviewed by a thoracic radiologist blinded to clinical outcomes. In cases of uncertainty, findings were discussed with additional radiologists. Interobserver agreement was not formally assessed. A representative thoracic CT scan is shown in **Figure 1**.

**Figure 1:**
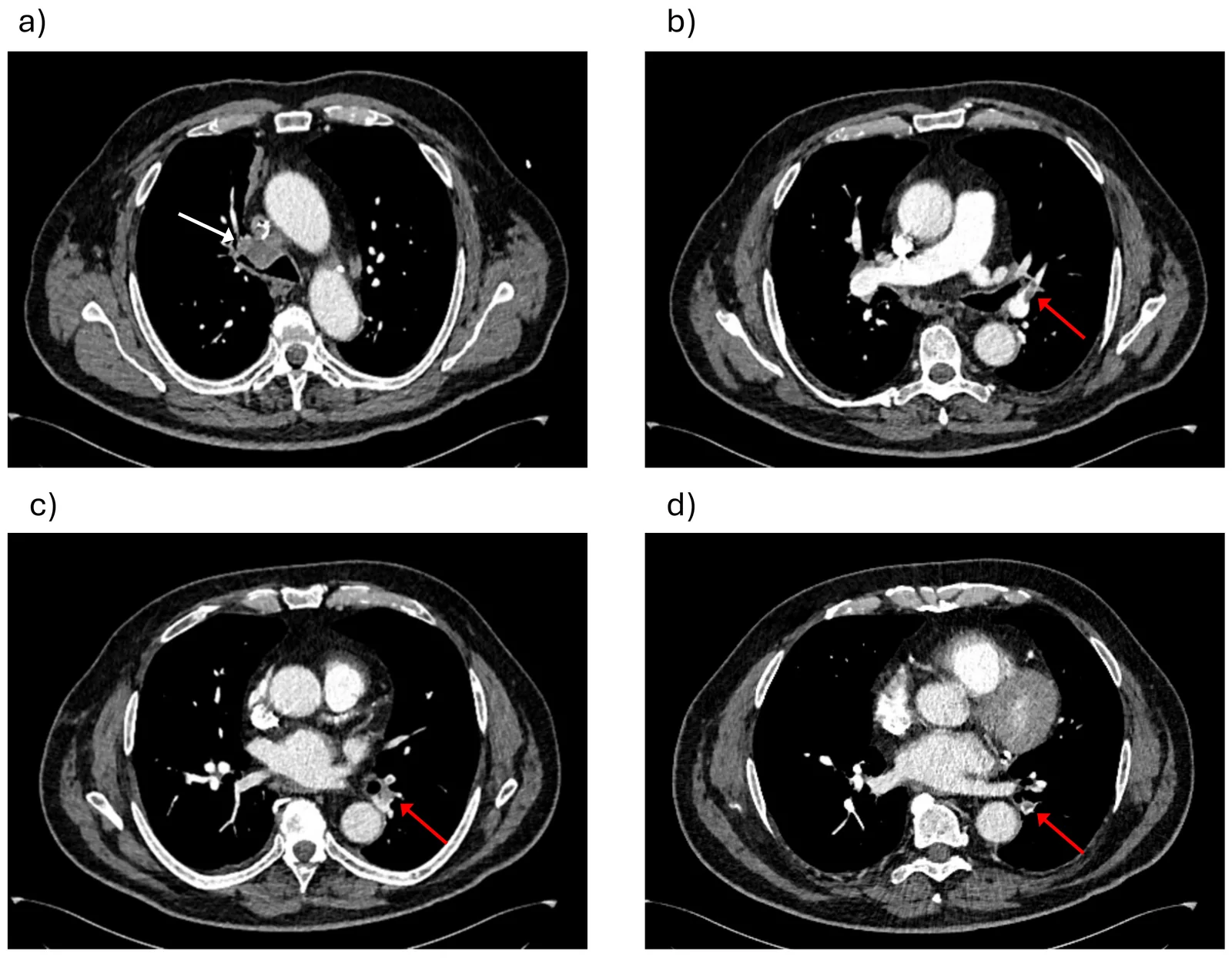
Representative contrast-enhanced CT demonstrating a subclinical pulmonary embolism. **(A)** Right central SCLC (white arrow); **(B)** - d) Segmental and subsegmental pulmonary emboli (red arrows) visualized on sequential axial CT pulmonary angiography images.

In our institution, all incidental pulmonary emboli, including isolated subsegmental pulmonary emboli, were treated with therapeutic anticoagulation after diagnosis in accordance with current national and international guideline recommendations for cancer-associated thrombosis (26, 27). Routine pharmacological thromboprophylaxis during outpatient systemic therapy was not standard practice at our institution and was reserved for selected indications, such as postoperative hospitalization or prolonged immobilization.Laboratory parameters, including C-reactive protein (CRP) and lactate dehydrogenase (LDH) were measured as part of routine clinical care in the hospital´s central laboratory using standardized automated assays according to the manufacturer´s instructions.

### Statistical analysis

Statistical analyses were performed for the overall cohort and separately for NSCLC and SCLC patients to account for biological and clinical heterogeneity between lung cancer subtypes. Continuous variables were assessed for normality and are presented as median and range; categorical variables are presented as absolute numbers and percentages. Group comparisons were conducted using Mann-Whitney U tests for continuous variables and chi-square or Fisher’s exact tests for categorical variables, as appropriate. Only patients with complete baseline data were included in the statistical analyses (complete-case analysis).

To identify independent predictors of subclinical PE, multivariable logistic regression analyses were performed for each cancer subtype. Variables, including age, Pack Years and BMI, were modeled as continuous predictors, while categorical variables were included as binary or multinomial factors as appropriate. Variables with a p-value < 0.10 in univariable analyses or with established clinical relevance were entered into the initial multivariable models. A backward elimination approach was applied to derive parsimonious final models. Model calibration was assessed using the Hosmer-Lemeshow goodness-of-fit test, discrimination by the area under the receiver operating characteristic curve (AUC), multicollinearity using variance inflation factors (VIFs) and events-per-variable (EPV) were calculated for the final models. Odds ratios (ORs) with 95% confidence intervals (CIs) were reported. We did not apply L1 or elastic net regression, as our aim was identification of clinically relevant risk factors. Given the moderate number of predictors relative to events, a conventional logistic regression approach was considered appropriate, allowing direct interpretation of effect estimates.

Overall survival was defined as the time from initiation of systemic chemo/(immuno) therapy to death or last follow-up. Survival was estimated using the Kaplan-Meier method and compared by log-rank test.

Cox proportional hazards models were used to assess the association between PE and survival. PE was modeled as a time-dependent covariate to account for its occurrence during follow-up and to avoid immortal time bias. Multivariable models were adjusted for age, BMI, ECOG performance status and disease stage. The proportional hazards assumption was assessed by testing interactions between the fixed covariates (age, BMI, ECOG performance status and disease stage) and log-transformed survival time. None of the interaction terms were statistically significant, indicating no evidence of violation of the proportional hazards assumption.

All statistical analyses were conducted using SPSS Statistics (IBM Corp., Armonk, NY). A p-value < 0.05 was considered statistically significant.

The study was conducted in compliance with the guidelines of the Institutional Review Board of the University Hospital Essen. The Ethics Committee of the University of Duisburg-Essen (Approval Number 23-11412-BO) waived the informed consent due to the retrospective and anonymous nature of this study. The data were completely anonymized before being included in the study.

## Results

### Patient characteristics

We included 373 patients diagnosed with either NSCLC or SCLC, who were treated between 2019 and 2022, including 179 women and 194 men. Of these patients, 261 had NSCLC and 112 had SCLC. Disease stage distribution included 22 patients with early-stage disease (I-IIB), 123 with locally advanced disease (IIIA-IIIC) and 228 with advanced-stage disease (IVA-IVB). Detailed baseline demographic and clinical characteristics are summarized in **Table 1**.

**Table 1:** Demographic and clinical characteristics at baseline.

Parameter	Median (Range) or Number (%)
Female/male, n (%)	179/194 (47/53)
Age, years	66 (22 – 86)
Body mass index, kg/m^2^	25 (12 – 46)
Smoking history, Pack Years	40 (0 – 150)
Stage	
I	6 (2)
II	16 (4)
IIIA	55 (15)
IIIB	50 (13)
IIIC	18 (5)
IVA	88 (24)
IVB	140 (38)
ECOG PS	
0	72 (20)
1	190 (51)
2	83 (22)
3	23 (6)
4	5 (1)
Histological Type	
Adenocarcinoma	146 (39)
Squamous Cell Carcinoma	86 (23)
Other NSCLC	29 (8)
Small Cell Carcinoma	112 (30)
Comorbidities	
Hyperlipidemia	114 (31)
Chronic Kidney Disease	11 (3)
Type 2 Diabetes	168 (45)
CAD/HF	243 (65)
Pulmonary Embolism	61 (16)
unilateral/bilateral	44/17
central	11 (18)
segmental	43 (71)
subsegmental	7 (11)
Systemic cancer treatments	
Cisplatin	295 (79)
Carboplatin	78 (21)
Taxane	181 (49)
Pemetrexed	98 (26)
Etoposide	110 (29)
Immunotherapy	240 (64)

Values are Median (Range) or Number (%).

ECOG PS, Eastern Cooperative Oncology Group performance status; NSCLC, Non-Small Cell Lung Cancer; SCLC, Small Cell Lung Cancer; CAD/HF, Coronary artery disease or heart failure.

### Pulmonary embolism

PE was diagnosed in 61 of the 373 patients, of whom 58 (95%) were clinically asymptomatic, yielding an overall incidence of 16.3% (**Figure 2**). Among the 58 subclinical PEs, 42 (72.4%) were detected during the first scheduled response assessment after two treatment cycles, whereas 16 (27.6%) were detected after four treatment cycles. In category-adjusted multivariable logistic regression analyses, male gender was significantly associated with a lower risk of PE in NSCLC patients (OR = 0.472; 95% CI: 0.233-0.958; p = 0.038, **Table 2**). In contrast, body mass index (BMI) was associated with an increased risk of PE (OR = 1.106; 95% CI: 1.030-1.197; p = 0.006, **Table 2**). In the final adjusted regression model, only BMI showed significant association with PE (OR = 1.111; 95% CI: 1.033-1.195; p = 0.005, **Table 3**).

**Figure 2:**
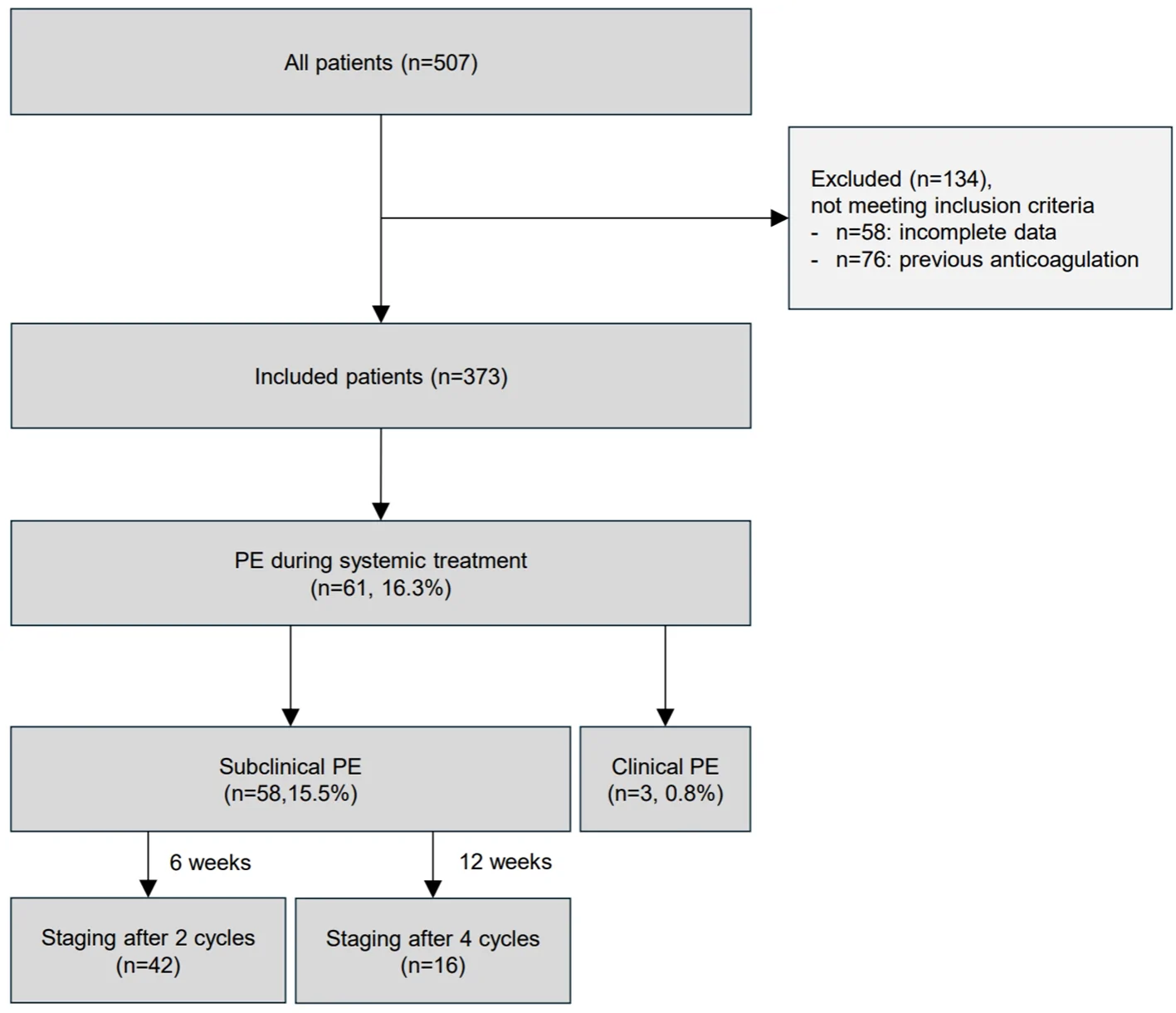
Patient flow chart. Of 507 patients treated between 2019 and 2022 and screened for study inclusion, 373 met the inclusion criteria and were included in the final analysis. Pulmonary embolism (PE) during systemic treatment was identified in 61 patients (16.3%), predominantly as subclinical events (n=58, 15.5%), whereas clinically manifest PE was rare (n=3, 0.8%). Subclinical PE was detected at routine staging after two (6 weeks) or four (12 weeks) treatment cycles.

**Table 2:** Category-adjusted multivariable logistic regression model for subclinical PE in NSCLC patients.

Clinical characteristics	Odds ratio	95% CI	p-value
Clinical characteristics			
Age	1.032	0.989 - 1.076	0.154
**Gender (male)**	**0.472**	**0.233 – 0.958**	**0.038**
Pack Years	0.988	0.971 – 1.005	0.164
ECOG PS	1.189	0.778 – 1.815	0.424
**BMI**	**1.106**	**1.030 - 1.197**	**0.006**
Comorbidities			
CAD/HF	0.949	0.461 – 1.954	0.887
DM	0.624	0.308 – 1.266	0.192
CKD	0.719	0.084 – 6.133	0.763
hyperlipidemia	1.521	0.713 – 3.244	0.278
Laboratory parameters			
CRP >1mg/dL	0.673	0.321 – 1.411	0.294
WBC >11/nL	1.320	0.584 – 2.983	0.504
HB <10g/dL	1.161	0.360 – 3.746	0.802
TC >300/nL	0.647	0.313 – 1.337	0.240
LDH >250U/L	0.720	0.352 – 1.471	0.368
Tumor characteristics			
Tumor stage IV vs. I-III	1.003	0.671 – 1.500	0.987
**Adenocarcinoma (vs. other NSCLC)**	**2.125**	**1.031 – 4.381**	**0.041**
Central vs. peripheral	0.611	0.249 – 1.502	0.283
Vascular infiltration	1.174	0.407 – 3.385	0.767
Bronchial stenosis	1.087	0.415 – 2.846	0.865
Treatment characteristics			
Cisplatin (vs. Carboplatin)	1.126	0.750-1.688	0.568
Immunotherapy	0.922	0.464-1.834	0.817
Biomarkers			
PD-L1	0.998	0.987-1.009	0.690

BMI, body mass index; ECOG PS, Eastern Cooperative Oncology Group performance status; CAD, coronary artery disease; HF, heart failure; DM, diabetes mellitus; CKD, chronic kidney disease; CRP, c-reactive protein; WBC, white blood cell count; HB, hemoglobin; TC, thrombocyte count; LDH, lactate dehydrogenase, NSCLC, non-small cell lung cancer. Statistical significance marked bold.

**Table 3:** Final adjusted regression model for subclinical PE in NSCLC patients.

Clinical characteristics	Odds ratio	95% CI	p-value
Final adjusted regression model			
Gender (male)	0.569	0.282-1.148	0.115
**BMI**	**1.111**	**1.033-1.195**	**0.005**
Adenocarcinoma (vs. other NSCLC)	2.083	0.989-4.385	0.063

BMI, body mass index. Statistical significance marked bold, NSCLC, non-small cell lung cancer.

Comorbidities including coronary artery disease and heart failure (CAD/HF), diabetes mellitus Type 2 (DM), chronic kidney disease and hyperlipidemia showed no statistically significant association with the development of PE in either NSCLC or SCLC (shown in **Tables 2**, **4**).

**Table 4:** Category-adjusted multivariable logistic regression model for subclinical PE in SCLC patients.

Clinical characteristics	Odds ratio	95% CI	p-value
Clinical characteristics			
Age	1.025	0.967 – 1.087	0.409
Gender (male)	0.700	0.245 – 2.005	0.498
Pack Years	1.004	0.974 – 1.035	0.793
ECOG PS	1.433	0.750 – 2.738	0.276
BMI	1.028	0.948 – 1.115	0.501
Comorbidities			
CAD/HF	1.019	0.288 – 3.375	0.982
DM	1.412	0.509 – 3.918	0.508
CKD	8.602	0.700 – 105.74	0.093
hyperlipidemia	1.452	0.487 – 4.332	0.504
Laboratory parameters			
CRP >1mg/dL	0.644	0.212 – 1.955	0.437
WBC >11/nL	1.761	0.570 – 5.439	0.326
HB <10g/dL	3.097	0.244 – 39.303	0.383
TC >300/nL	0.983	0.336 – 2.877	0.975
**LDH >250U/L**	**3.750**	**1.015 – 13.860**	**0.048**
Tumor characteristics			
Tumor stage IV vs. I-III	1.476	0.762 – 2.859	0.248
**Central vs. peripheral**	**6.688**	**1.010 – 44.265**	**0.049**
**Vascular infiltration**	**7.296**	**1.531 – 34.763**	**0.013**
Bronchial stenosis	0.921	0.219 – 3.870	0.911
Treatment characteristics			
Cisplatin (vs. Carboplatin)	1.564	0.472 – 5.184	0.465
Etoposide (vs. taxanes)	2.918	0.347 – 24.565	0.325
Immunotherapy	0.922	0.311 – 2.733	0.884
Biomarkers			
NSE.	0.999	0.994-1.003	0.524
Ki-67	0.996	0.944-1.052	0.894

BMI, body mass index; ECOG PS, Eastern Cooperative Oncology Group performance status; CAD, coronary artery disease; HF, heart failure; DM, diabetes mellitus; CKD, chronic kidney disease; CRP, c-reactive protein; WBC, white blood cell count; HB, hemoglobin; TC, thrombocyte count; LDH, lactate dehydrogenase; NSE, neuron-specific enolase. Statistical significance marked bold.

Multiple serum- and biomarkers were analyzed for their association with PE. As indicated in **Table 2**, none of the tested parameters in NSCLC, including C-reactive protein (CRP), leukocyte count, hemoglobin, platelet count, lactate dehydrogenase (LDH) and PD-L1 were significantly associated with PE. In contrast, in the SCLC subgroup, elevated LDH levels > 250 U/L were significantly associated with PE in category-adjusted analysis (OR = 3.750; 95% CI: 1.015-13.860; p = 0.048, **Table 4**), but lost its significance in the final model (**Table 5**).

**Table 5:** Final adjusted regression model for subclinical PE in SCLC patients.

Clinical characteristics	Odds ratio	95% CI	p-value
Final adjusted regression model			
LDH >250U/L	2.413	0.700 – 8.315	0.163
**Central vs. peripheral**	**5.637**	**1.206 – 26.349**	**0.028**
**Vascular infiltration**	**7.683**	**1.628 – 36.259**	**0.010**

LDH, lactate dehydrogenase.

With regard to tumor-related parameters, in NSCLC patients, adenocarcinoma histology was significantly associated with an increased risk of PE compared to other histotypes (OR = 2.125; 95% CI: 1.031-4.381; p = 0.041, **Table 2**), but this association did not remain statistically significant in the final adjusted model (**Table 3**). Other tumor-specific variables including stage, location (central vs. peripheral), vascular infiltration and bronchial stenosis, were not significantly associated. The final NSCLC model demonstrated adequate calibration (Hosmer-Lemeshow χ² = 11.603, df = 8, p = 0.170) and modest discrimination (AUC = 0.664). No evidence of multicollinearity was observed (all VIFs ≤ 1.03). The final model included three predictors with 41 PE events, corresponding to an EPV of 13.7.

In SCLC patients, central tumor location showed a strong association with PE (OR = 6.688; 95% CI: 1.010-44.265; p = 0.049, **Table 4**), as did vascular infiltration (OR = 7.296; 95% CI: 1.531-34.763; p = 0.013, **Table 4**). Both factors remained statistically significant in the final adjusted regression model (**Table 5**). The final SCLC model also demonstrated adequate calibration (Hosmer-Lemeshow χ² = 7.295, df = 8, p = 0.505) and acceptable discrimination (AUC = 0.730). No evidence of multicollinearity was observed (all VIFs ≤ 1.09). The final model included three predictors with 20 PE events, corresponding to an EPV of 6.7.

### Systemic treatment

No significant differences in systemic treatment distribution were observed between patients with and without PE, as detailed in **Table 6**. In both, NSCLC and SCLC patients, no treatment-related associations were identified (**Tables 3**–**5**).

**Table 6:** Clinical characteristics of patients with/without pulmonary embolism.

Parameter	PE	No PE	p-value
Clinical characteristics	n=61	n=312	
Female/male, n (%)	35/26 (57/43)	144/168 (46/54)	0.123
Age, years	67 (22-85)	65 (25-86)	0.056
**Body mass index, kg/m^2^**	**26.58 (12 – 46)**	**24.98 (16 – 45)**	**0.019**
Smoking history (Pack Years)	40 (0 – 90)	40 (0-150)	0.467
ECOG PS			
0	8	66	
1	31	157	
2	17	66	0.321
3	3	20	
4	2	3	
TNM			
T1	14	42	
T2	9	40	
T3	13	54	0.104
T4	25	176	
N+	55	256	0.297
N1	8	35	
N2	25	114	0.310
N3	22	107	
M+	42	187	
M1a	12	42	
M1b	5	29	0.470
M1c	25	116	
Tumor Characteristics			
NSCLC/SCLC	41/20	220/92	0.791
**central/peripheral**	**23/38**	**102/210**	**0.05**
**vascular infiltration**	**51**	**209**	**0.007**
bronchial stenosis	31	180	0.052
Treatment			
Cisplatin	54 (89)	241 (77)	0.157
Carboplatin	7 (11)	71 (23)	0.06
Taxane	25 (41)	156 (50)	0.182
Pemetrexed	22 (36)	76 (24)	0.08
Etoposid	20 (33)	90 (31)	0.544
Immunotherapy	39 (64)	201 (64)	0.883

Values are Median (Range) or Number (%).

ECOG PS, Eastern Cooperative Oncology Group performance status; NSCLC, Non-Small Cell Lung Cancer; SCLC, Small Cell Lung Cancer. Statistical comparisons between groups were calculated with chi-squared-test and Mann-Whitney-Test. Statistical significance marked bold.

PE (including both subclinical and clinically manifest events) was associated with significantly impaired overall survival. Kaplan-Meier analysis demonstrated a marked separation of survival curves, with patients with PE exhibiting substantially shorter survival compared with patients without PE (log-rank test χ² = 10.11, p = 0.001). Median overall survival was 14.6 months (95% CI 5.7-23.4) in patients with PE compared with 24.0 months (95% CI 20.8-27.2) in those without PE (**Figure 3A**). In multivariable Cox regression adjusted for age, BMI, ECOG performance status and disease stage, PE remained independently associated with worse overall survival (HR 1.54, 95% CI 1.11-2.13; p = 0.010).

**Figure 3:**
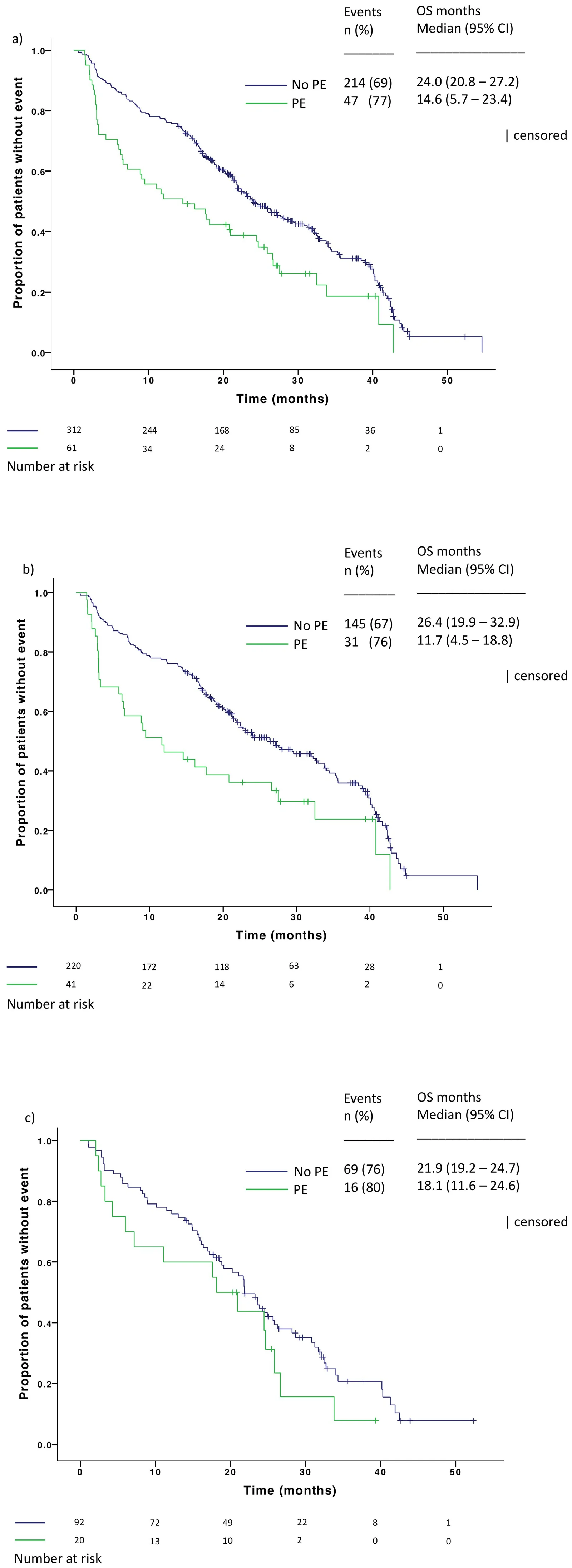
Kaplan-Meier overall survival curves according to PE status. Tick marks indicate censored observations (patients alive at their last follow-up). Numbers at risk are displayed below the x-axis at 0, 10, 20, 30, 40, and 50 months. **(A)** Overall survival; **(B)** survival in patients with NSCLC; **(C)** survival in patients with SCLC.

ECOG performance status (HR 1.35, p < 0.001) and stage IV disease (HR 1.43, p < 0.001) were also independent predictors of mortality, whereas age and BMI were not (**Supplementary Table 1**).

In patients with NSCLC, PE was associated with significantly worse overall survival. Kaplan-Meier curves demonstrated early and sustained differences between groups, and log-rank testing confirmed a significant difference in survival distributions (χ² = 8.55, p = 0.003). Median survival was 11.7 months (95% CI 4.5-18.8) in NSCLC patients with PE compared with 26.4 months (95% CI 19.9-32.9) in those without PE (**Figure 3B**). Accordingly, in multivariable Cox analysis, PE remained an independent predictor of mortality in NSCLC patients (HR 1.81, 95% CI 1.21-2.70; p = 0.004) (**Supplementary Table 2**).

In contrast, among patients with SCLC, no significant difference in overall survival was observed between patients with and without PE. Median survival time was 18.1 months (95% CI 11.6-24.6) in patients with PE and 21.9 months (95% CI 19.2-24.7) in those without PE, and Kaplan-Meier curves largely overlapped (**Figure 3C**). Log-rank analysis confirmed the absence of a statistically significant survival difference (χ² = 1.88, p = 0.170). Consistently, PE was not significantly associated with overall survival in multivariable analysis in the SCLC subgroup (HR 1.17, 95% CI 0.66-2.06; p = 0.592, **Supplementary Table 3**).

## Discussion

In this retrospective single-center study including 373 patients with lung cancer treated between 2019 and 2022, PE was detected in 16.3% (n=61) of patients, of whom 95% (58/61) were subclinical, underscoring the substantial burden of asymptomatic thromboembolic disease in this population. Importantly, our analysis revealed distinct and histology-specific risk profiles for PE under systemic induction therapy in NSCLC and SCLC, possibly due to the different thrombogenic mechanisms between these histologic subtypes.

Interestingly, our data demonstrated a marked discrepancy between the high incidence of subclinical PE and the low rate of clinically manifest events during induction therapy.

In a meta-analysis Chen et al. reported PE incidence rates in lung cancer patients of 8.3% (28).

This discrepancy may be explained by frequent imaging during systemic therapy, leading to increased detection of incidental PE, as well as early-stage thromboembolic events before the development of clinical symptoms.

The relatively high incidence of PE observed in our cohort likely reflects, at least in part, the standardized imaging protocol with scheduled CT-based response assessments. Unlike routine clinical practice, where imaging is performed less frequently and primarily in response to clinical symptoms or suspicion, our surveillance strategy facilitated the detection of otherwise asymptomatic PE. Consequently, the observed incidence is likely influenced by surveillance intensity and should not necessarily be interpreted as indicating a higher underlying thromboembolic risk compared with cohorts undergoing less frequent imaging.

In NSCLC, male gender was initially associated with reduced PE risk, but this association did not persist after multivariable adjustment, indicating that gender was not an independent risk factor. This finding aligns with larger cohort studies which failed to identify gender as an independent predictor of thromboembolic events in lung cancer (18).

Although adenocarcinoma histology was associated with PE in multivariable analyses, this effect was attenuated in the final adjusted model, indicating that histological subtype alone may not fully drive thromboembolic risk once patient-related and clinical factors are taken into account. Nevertheless, the observed association is consistent with extensive prior evidence demonstrating the heightened thrombogenicity of adenocarcinoma. Adenocarcinoma cells are known to express higher levels of tissue factor and mucins, promoting coagulation activation and platelet-endothelial interactions that contribute to systemic hypercoagulability. These biological features are epidemiologically supported by large cohort studies, consistently identifying adenocarcinoma histology as a major determinant of venous thromboembolism risk across solid tumors (3, 19, 29, 30).

In contrast, SCLC demonstrated a predominantly morphology- and anatomy-driven risk profile. In the adjusted regression model, central tumor location and vascular infiltration emerged as independent predictors of PE, indicating a particularly high embolic risk. These observations suggest that in SCLC, aggressive local tumor characteristics and direct vascular involvement may contribute substantially to thromboembolic risk. Prior reports describing PE in the setting of tumor mass effect or intravascular extension further corroborate the relevance of local anatomical factors in highly aggressive malignancies (19, 31).

Elevated lactate dehydrogenase (LDH) was associated with increased PE risk in SCLC in category-specific multivariable analyses, but not after full adjustment, indicating that LDH reflects tumor aggressiveness rather than representing an independent risk factor. As a well-established surrogate marker of tumor burden and aggressive tumor biology, particularly in SCLC, LDH is associated with increased cellular turnover, hypoxia and necrosis (32). Supporting this rationale, Gizzi et al. demonstrated that elevated LDH independently predicted thromboembolic events in patients with highly proliferative germ cell tumors undergoing cisplatin-based chemotherapy (33). Thus, LDH may reflect aggressive tumor biology associated with thromboembolic risk in SCLC, although its independent predictive value appears attenuated.

In NSCLC, increased BMI was the only factor that remained independently associated with PE in the final regression model, consistent with established evidence linking BMI to cancer-associated thrombosis (17, 34). The persistence of BMI after full multivariable adjustment highlights the relevance of patient-related metabolic factors in cancer-associated PE beyond tumor-specific characteristics. Obesity promotes a prothrombotic milieu through chronic low-grade inflammation, endothelial dysfunction, elevated levels of procoagulant factors and impaired fibrinolysis (35). These mechanisms underpin the inclusion of BMI in the Khorana risk score, originally described by Khorana et al. and align with findings by Hiraide et al., who reported a more than two-fold increased risk of thromboembolic events in lung cancer patients with elevated BMI (36, 37).

Notably, traditional cardiovascular and metabolic comorbidities, including coronary artery disease and type 2 diabetes mellitus, were not significantly associated with PE in either cancer subtype in our cohort. This contrasts with population-based studies identifying heart disease and type 2 diabetes mellitus as independent venous thromboembolism risk factors (38, 39). This discrepancy possibly reflects the predominance of tumor- and treatment-related mechanisms over patient-related comorbidities in cancer-associated thrombosis. The relatively high prevalence of metabolic comorbidities likely reflects the advanced age and substantial cardiovascular burden of a real-world cohort in the German Ruhr Area.

Cisplatin-based chemotherapy and ICI therapy were not significantly associated with PE in our cohort, despite prior reports linking both modalities to clinically overt venous thromboembolism (19–21, 24). This discrepancy may reflect differences in outcome definitions, as our study focused specifically on asymptomatic PE detected on routine imaging, suggesting that treatment-related thrombogenicity may manifest differently in subclinical versus symptomatic settings.

Our survival analyses demonstrated a significant association between PE and reduced overall survival in patients with lung cancer, predominantly driven by the NSCLC subgroup. In both the overall cohort and the NSCLC subgroup, PE was associated with markedly reduced overall survival and early separation of Kaplan-Meier curves. Importantly, this association should not be interpreted as evidence of a causal relationship. Rather, PE may represent a surrogate marker of aggressive tumor biology, advanced disease, heightened hypercoagulability or other unmeasured factors that collectively contribute to poor prognosis. This suggests that PE may reflect aggressive tumor biology, heightened hypercoagulability or advanced disease rather than representing solely an acute clinical complication. Similar observations have been reported in pancreatic cancer, where venous thromboembolism was independently associated with reduced overall survival during perioperative therapy (40).

Survival analyses were based on overall PE events, as clinically manifest PE was rare; notably, 95% of events were subclinical, indicating that the observed survival associations are largely driven by subclinical PE.

In contrast, PE did not significantly affect survival in patients with SCLC, potentially reflecting the intrinsically aggressive disease course and uniformly poor prognosis of this subtype, which may outweigh the prognostic relevance of thromboembolic events.

Several limitations must be acknowledged. The retrospective, single-center design limits generalizability and introduces potential selection and documentation bias. Despite adjustment for relevant clinical parameters, residual confounding cannot be excluded because several established thrombosis-associated variables were unavailable. Lack of detailed stratification by treatment timing relative to PE onset constrains causal interpretation of therapy-related risk. Some associations identified in the SCLC subgroup were accompanied by wide confidence intervals, reflecting the limited number of PE events and the resulting reduced statistical precision. Accordingly, these findings should be considered exploratory and require confirmation in larger independent cohorts.

Given the retrospective and exploratory nature of the present study, backward selection was used to identify candidate predictors. Validation in larger, independent cohorts using prespecified clinically informed models or penalized regression techniques will further strengthen the robustness and generalizability of these findings. Additionally, D-dimer levels were not consistently available, precluding evaluation of this well-established biomarker for thromboembolic screening.

Although the NSCLC model met conventional recommendations for events-per-variable, the lower number of PE events in the SCLC subgroup resulted in a lower EPV, which may have reduced the stability of subgroup-specific estimates despite satisfactory calibration and the absence of multicollinearity.

Key strengths of this study include its real-world design, inclusion of both major lung cancer subtypes across all disease stages and evaluation of contemporary treatment regimens. The relatively high incidence of subclinical PE enabled meaningful subgroup analyses and provided novel insights into asymptomatic thromboembolic risk.

Our findings support a tailored approach to PE risk stratification in lung cancer patients. In NSCLC, BMI emerged as the dominant independent risk factor, whereas in SCLC, anatomical tumor characteristics were the principal determinants of thromboembolic risk. These results argue against a one-size-fits-all strategy and highlight the need for histology-specific risk assessment models for cancer-associated thrombosis. Although BMI is incorporated in established risk scores such as the Khorana model, its persistence in the regression analyses suggests particular relevance in NSCLC and supports histology-adapted refinement of existing risk stratification tools. Enhanced vigilance for subclinical PE may be warranted, particularly in high-risk subgroups, to inform individualized surveillance and thromboprophylaxis strategies.

In summary, this study demonstrates a high incidence of subclinical PE in lung cancer patients and reveals marked heterogeneity in thromboembolic risk factors between NSCLC and SCLC. Importantly, PE was associated with significantly reduced overall survival, particularly in patients with NSCLC, underscoring its value as a prognostic marker rather than implying a direct causal effect on mortality. Tumor biology, anatomical features, metabolic activity and treatment exposures differentially influence PE risk across histological subtypes. These results emphasize the clinical relevance of subclinical PE and support the development of personalized, histology-specific approaches to thromboembolic risk assessment and management in lung cancer.

## Data Availability

The data analyzed in this study is subject to the following licenses/restrictions: The datasets analyzed during the current study are not publicly available due to patient confidentiality and ethical restrictions. Requests to access these datasets should be directed to johannes.wienker@rlk.uk-essen.de.

## Supplementary material

Supplementary Table 1 Multivariable Cox proportional hazards regression analysis for overall survival. Hazard ratios (HRs) with 95% confidence intervals (CIs) are shown. The model was adjusted for age, body mass index, ECOG performance status and disease stage.

Supplementary Table 2 Multivariable Cox proportional hazards regression analysis for survival in NSCLC patients. Hazard ratios (HRs) with 95% confidence intervals (CIs) are shown. The model was adjusted for age, body mass index, ECOG performance status and disease stage.

Supplementary Table 3 Multivariable Cox proportional hazards regression analysis for survival in SCLC patients. Hazard ratios (HRs) with 95% confidence intervals (CIs) are shown. The model was adjusted for age, body mass index, ECOG performance status and disease stage.
