# Supplementary material for: Subclinical pulmonary embolism in first-line/induction therapy of lung cancer - a remaining clinically relevant adverse event
Source: Front Oncol. 2026 Sep 21;16:1886848. doi: 10.3389/fonc.2026.1886848 (PMC13635988; doi:10.3389/fonc.2026.1886848)
Supplement: Supplementary file 1 [file Table1.pdf]

	HR	95% CI	p-value
**Pulmonary embolism**	**1.54**	**1.11 – 2.13**	**0.01**
Age	1.01	0.99 – 1.02	0.508
BMI (kg/m^2)	1.01	0.98 – 1.03	0.536
**ECOG PS**	**1.35**	**1.16 – 1.58**	**<0.001**
**Stage IV**	**1.43**	**1.24 – 1.65**	**<0.001**
