# Supplementary material for: Subclinical pulmonary embolism in first-line/induction therapy of lung cancer - a remaining clinically relevant adverse event
Source: Front Oncol. 2026 Sep 21;16:1886848. doi: 10.3389/fonc.2026.1886848 (PMC13635988; doi:10.3389/fonc.2026.1886848)
Supplement: Supplementary file 2 [file Table2.pdf]

	HR	95% CI	p-value
**Pulmonary embolism**	**1.807**	**1.211– 2.696**	**0.004**
Age	1.005	0.99 – 1.02	0.541
BMI (kg/m^2)	0.996	0.96 – 1.03	0.800
**ECOG PS**	**1.38**	**1.16 – 1.66**	**<0.001**
**Stage IV**	**1.42**	**1.2 – 1.69**	**<0.001**
