# Supplementary material for: Subclinical pulmonary embolism in first-line/induction therapy of lung cancer - a remaining clinically relevant adverse event
Source: Front Oncol. 2026 Sep 21;16:1886848. doi: 10.3389/fonc.2026.1886848 (PMC13635988; doi:10.3389/fonc.2026.1886848)
Supplement: Supplementary file 3 [file Table3.pdf]

	HR	95% CI	p-value
Pulmonary embolism	1.17	0.661– 2.063	0.592
Age	1.006	0.98 – 1.03	0.646
BMI (kg/m^2)	1.026	0.99 – 1.07	0.172
ECOG PS	1.35	0.992 – 1.84	0.056
**Stage IV**	**1.44**	**1.1 – 1.88**	**0.007**
